# Acute and Reversible Hypothalamic Symptoms in a Lateral Head Impact Mouse Model of Mild Traumatic Brain Injury

**DOI:** 10.1089/neur.2024.0071

**Published:** 2024-08-08

**Authors:** Julie O’Reilly-Fong, Nick J. Simpson, Zahra S. Thirouin, Paolo A. Bastone, Cristian Zaelzer, Anzala Murtaz, Charles W. Bourque

**Affiliations:** Brain Repair and Integrative Neuroscience Program, Research Institute of the McGill University Health Center, Montreal, Canada.

**Keywords:** c-Fos, hypothalamus, hypothermia, traumatic brain injury

## Abstract

Central autonomic and endocrine dysfunctions following traumatic brain injury (TBI) are believed to involve the hypothalamus; however, underlying mechanisms are unknown. Although chronic deficits might be caused by irreversible tissue damage, various neuroendocrine and autonomic symptoms are only observed transiently, suggesting they might result from a temporary alteration in the activity of hypothalamic neurons. We therefore examined if a mouse model of mild TBI could induce reversible autonomic phenotypes and cause acute changes in c-Fos expression within corresponding regions of the hypothalamus. Adult C57Bl/6 male mice were lightly anesthetized with isoflurane and subjected to TBI by lateral head impact using a Gothenburg impactor. Mice treated the same way, but without the head impact served as controls (shams). We monitored body weight and core body temperature by infrared thermography and performed immunohistochemistry against c-Fos in various regions of the hypothalamus. We determined that a projectile velocity of 9 m/s significantly delayed recovery from the anesthesia without inducing skull fractures and signs of discomfort disappeared within 3 h, as assessed by grimace scale. Compared with shams, TBI mice displayed a rapid decrease in core body temperature which resolved within 48 h. Daily body weight gain was also significantly lower in TBI mice on the day following injury but recovered thereafter. c-Fos analysis revealed a significantly higher density of c-Fos-positive cells in the paraventricular nucleus and a significantly lower density in the median preoptic nucleus and medial preoptic area. We conclude that mild TBI induced by a single lateral head impact in mice at 9 m/s produces acute and reversible symptoms associated with hypothalamic dysfunction accompanied by significant changes in c-Fos expression within relevant areas of the hypothalamus. These findings support the hypothesis that a temporary alteration of neuronal activity may underlie the expression of reversible central autonomic and neuroendocrine symptoms.

## Introduction

Traumatic brain injury (TBI) is defined as an alteration in brain function caused by an external force.^1^ Nonpenetrating TBI typically follows a blunt impact to the head induced mainly by unintentional falls, traffic accidents, and sports injuries.^2^ Acute symptoms that follow TBI are most commonly described by the nature of the motor and cognitive deficits they produce.^3,4^ However, patients who have suffered TBI can also present with a broad spectrum of other symptoms including autonomic^5–9^ and neuroendocrine^10–13^ dysfunctions. For example, defects in thermoregulation,^14,15^ abnormal pupillary responses,^16^ and impaired heart rate variability^17^ are frequently observed following TBI. TBI patients can also display disturbances in eating behavior,^18,19^ cortisol profiles,^20^ and electrolyte balance,^21^ which may reflect alterations in neuroendocrine function.

The hypothalamus is well-known to control autonomic and neuroendocrine processes.^22^ Therefore, post-TBI deficits in these processes are commonly attributed to hypothalamic or pituitary injury.^23^ Although brain injury is commonly recognized as irreversible, many of the symptoms mentioned above are only observed acutely and resolve within days or weeks in both humans^11,24^ and animal models.^25,26^ This observation implies that such symptoms do not reflect irreversible tissue damage but a temporary alteration in neuronal or circuit function. Indeed, in addition to physical injury caused by mechanical force acting on cerebral tissue, TBI can trigger pathophysiological processes including mitochondrial dysfunction, oxidative stress, and neuroinflammation^27^ that could temporarily alter the activity of hypothalamic neurons. However, it remains unclear how TBI could induce such pathophysiological effects in the absence of tissue injury. Previous work has shown that mechanosensitive ion channels could mediate the activation or inactivation of neuronal activity^28^ as well as calcium-dependent signaling cascades that could underlie prolonged effects. It would therefore be valuable to develop a model of TBI that could be used for the analysis of robust and acute hypothalamic phenotypes, together with an examination of neuronal activity in relevant hypothalamic nuclei.

In principle, the functional state of the brain immediately after TBI could be investigated using changes in the expression of c-Fos, an immediate early gene whose calcium-dependent expression rises in response to a variety of stimuli.^29^ Indeed, previous studies using various models of TBI in rodents have shown widespread c-Fos expression in many brain regions,^30–32^ including in the hypothalamus.^33^ The objective of this study was specifically to characterize c-Fos expression in the hypothalamus of a mouse model of mild TBI that does not feature invasive wounds or skull fractures, yet presents a reversible hypothalamic phenotype.

## Materials and Methods

### Mice

Animal procedures were conducted in accordance with the guidelines outlined by the Canadian Council on Animal Care (http://www.ccac.ca/), and experiments adhered to protocols approved by the Facility Animal Care Committee of McGill University (animal use protocol 1190). Mice were bred and housed under standard conditions (12:12 h light-dark cycle; food and water *ad libitum*). All experiments were performed on 2–4-month-old male C57Bl/6 mice obtained from Charles River Laboratories.

### Traumatic brain injury

TBI was administered using a Gothenburg impactor (Collision Analysis, Calgary, Alberta, Canada)^34^ to produce a single lateral head impact in mice. Each mouse was briefly anesthetized (∼15 s) with 5% isoflurane in an induction chamber and immediately placed in a prone position on a Teflon^®^ surface with the left side of the head resting against an aluminum plate (35 × 14 mm and 3 mm thick; “helmet”). Using pneumatic pressure, a 50 g projectile was propelled through a barrel and contacted the helmet which distributed the force of the impact to the lateral surface of the mouse’s head, causing unrestrained rotational acceleration. Sham mice were treated the same way but placed outside the impactor when the projectile was fired (to expose the mouse to the sounds of the procedures). After TBI or sham treatment, mice were placed in a supine position in their home cages, and the time taken to restore a prone position was recorded (time-to-right) as a measure of unconsciousness. All TBIs were performed with a projectile velocity of 9 m/s unless specified otherwise.

### Grimace scale

TBI mice were evaluated for facial expression of pain using the grimace scale.^35^ Thirty minutes before TBI, immediately before TBI, and 1, 5, 10, 15, 30, 60, 120, and 180 min after TBI, an evaluator carefully observed each mouse and assigned a value of 0 (absent), 1 (moderately present), or 2 (severe) for the following five features: orbital tightening, nose bulge, cheek bulge, ear position, and whisker change. The grimace scale score was then calculated as the average of the five values.

### Infrared thermography

Temperature measurements were obtained using a thermal camera (E85: 384X288 Thermal Camera, Teledyne FLIR, Oregon, USA) with a 42° lens (FLIR). Before imaging, cages were placed under the thermal camera and the lid and nesting material were removed. Recordings were conducted in a paired fashion (one TBI and one sham mouse in adjacent cages) and the position of the cages (left or right) was inversed in consecutive sessions. A first cohort of mice was used for evaluating the immediate effect of TBI on core body temperature (measured as eye temperature^36^) brown adipose tissue (BAT) and tail temperature. One day before the experiment, the upper dorsal aspect of each mouse was shaved to obtain accurate BAT temperature values. Mice were habituated to the setup for 1.5 h, after which thermal images were recorded for 45 min as baseline. Mice were then subjected to sham or TBI and immediately returned to their cage under the camera to collect another 45 min of thermal data. All TBI/sham treatments were done at Zeitgeber time (ZT) 18. In another cohort of mice, core body temperature was monitored for 3 days before and 6 days after TBI/sham (day 0). Recordings lasted 15 min and were performed at the same time every day (ZT20). On day 0, TBI/sham was performed 2 h before recording the mouse. Thermography videos were analyzed using FLIR Tools v6.4 (Teledyne FLIR). For each frame analyzed, a 9-pixel region of interest was centered over the BAT, eye, or tail to register the maximal average temperature at each site. For the short-term experiment, a representative frame was selected in each 5 min time bin. Baseline values were calculated as the average temperature over the last 20 min before TBI/sham. After TBI/sham, the change in temperature was expressed as the average of three adjacent values resulting in the maximal change relative to baseline. For the long-term temperature analysis, daily core body temperature was expressed as the average eye temperature observed each minute during the 15-min session.

### Weight

Mice were weighed 1 min before and up to 15 days after TBI/sham, at the same time every day (ZT18-ZT19). Change in body weight was calculated as the change in weight relative to the day of TBI/sham. Daily weight gain was expressed as the change in weight relative to the previous day.

### Immunohistochemistry

One hour after TBI/sham, mice were anesthetized with isoflurane and perfused transcardially with phosphate-buffered saline (PBS) containing 4% paraformaldehyde (#158127, MilliporeSigma Canada Ltd., Oakville, ON, Canada) at room temperature. Brains were extracted and immersed in the same solution for at least 24 h at 4°C, then cut into 50 µm-thick serial sections with a vibratome (VT1200, Leica Biosystems Inc., Concord, ON, Canada) in the coronal plane. Selected hypothalamic sections were blocked with 10% normal goat serum (#G9023, MilliporeSigma Canada Ltd.) in PBS containing 0.3% Triton (#X-100, MilliporeSigma Canada Ltd.) for 1 h at room temperature, washed three times in PBS + 0.3% Triton, and incubated for 24 h at 4°C with a rabbit polyclonal primary antibody directed against c-Fos (1:2,000, #226008, Synaptic Systems GmbH, Göttingen, Germany). Following three washes, sections were incubated for 2 h at room temperature with fluorescently labeled secondary antibodies goat anti-rabbit Alexa fluor 568 (1:500, #A11011, Thermo Fisher Scientific Inc., Waltham, MA, USA). Sections were then washed and mounted on glass slides using Prolong Gold Antifade mounting media (#P36930, Thermo Fisher Scientific Inc.).

### Image acquisition and analysis

Images were collected using a confocal microscope (FV3000, Olympus Canada, Richmond Hill, ON, Canada) with a 10× objective. Coronal slices selected for analysis were situated at anteroposterior coordinates +0.5, +0.6, −0.7, −1.2, and −2.3 mm relative to Bregma. Regions of interest were defined following the third edition of The Mouse Brain atlas published by Franklin and Paxinos.^37^ Image analysis was performed using Fiji (v1.54i; http://imagej.org). For each image, a median filter (2.0 pixel) was applied, regions of interest were delineated using the elliptical or polygon selection tool, and the area was recorded. The c-Fos signal in each region of interest was manually thresholded until background was eliminated. The watershed function was applied to the binary image before c-Fos nuclei were counted using the analyze particles tool with a 25-infinity µm^2^ size filter. The number of nuclei was then normalized by area to express c-Fos density per 0.1 mm^2^.

### Statistics

Statistical analyses were performed using SigmaPlot 12.3 (Systat Software Inc., Chicago, IL, USA). Tests used are indicated in the text. Nonparametric tests were applied when recommended by the software. All data are expressed as mean ± standard error of the mean (s.e.m.). Differences were considered significant when *p* < 0.05.

## Results

**FIG. 1.**
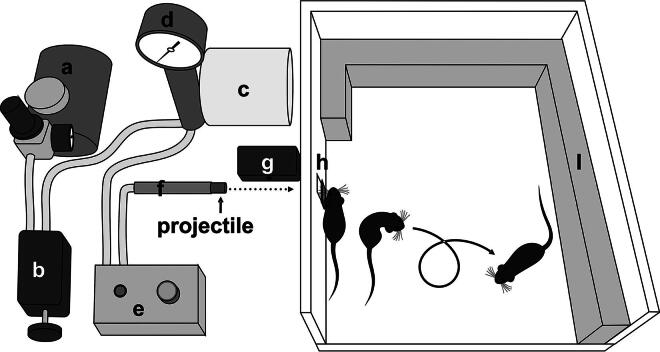
Simplified schematic of the lateral head impactor. Compressed air is generated by the compressor **(a)**. The user defines via the regulator **(b)** the amount of pressure accumulated in the air tank **(c)**, which can be read on the pressure gauge **(d)**. When the switch **(e)** is activated, the pressure is released into the cannon, **(f)** which propels the projectile. The speed is recorded by the velocimeter **(g)**. The projectile lands on the helmet **(h)** which distributes the force of the impact to the head of a lightly anesthetized mouse. A foam cushion **(i)** is placed in the direction of the mouse’s movement.

We adapted the model developed by Viano and colleagues^34^ where a Gothenburg device is used to deliver a calibrated impact to the left side of the head of a lightly anesthetized mouse (Fig. 1). The force of the impact is proportional to the velocity of a 50 g projectile and is distributed to the head of the mouse via an intermediary “helmet.” This approach avoids external lesions and promotes a rotational acceleration of the head which represents a key pathogenic cause of injury in human TBI.^38^

**FIG. 2.**
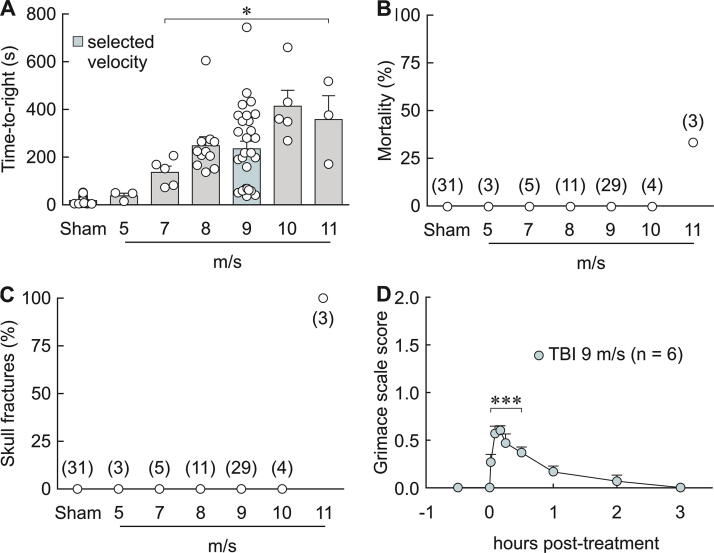
Lateral head impact with a projectile velocity of 9 m/s produced TBI without external injury or prolonged pain. **(A)** Time-to-right in shams and TBI with different projectile velocities. Dots are individual values. *p < 0.05, one-way ANOVA on ranks followed by Dunn’s test. **(B)** Lateral head impact does not cause mortality below a velocity of 11 m/s. The number of mice used to calculate percent mortality is indicated in parentheses for each group. **(C)** Lateral head impact does not cause skull fractures below a velocity of 11 m/s. The number of mice used to calculate percent skull fractures is indicated in parentheses for each group. **(D)** Lateral head impact at 9 m/s induces a temporary grimace scale score > 0 (*p* = 0.003 at 1 min and *p* < 0.001 at 5, 10, 15, and 30 min) that subsided by 3 h after injury. ***p < 0.001, one-way RM ANOVA followed by Holm Sidak vs. 30 min before injury. TBI, traumatic brain injury; ANOVA, analysis of variance.

As illustrated in Figure 2A, lightly anesthetized animals that were not subjected to TBI (sham) but placed in a supine position re-established a mean (± s.e.m.) prone mobile position (time-to-right) of 15.8 ± 2.4 s. In contrast, animals subjected to TBI displayed a progressively longer time-to-right compared with shams as the velocity of the projectile was increased (one-way ANOVA on ranks H[6] = 57.82, *p* < 0.001 followed by Dunn’s multiple comparisons vs. sham: 5 m/s 41.0 ± 13.2 s, *p* = ns; 7 m/s 145.3 ± 22.7 s, *p* < 0.05; 8 m/s 247.6 ± 38.3 s, *p* < 0.05; 9 m/s 235.3 ± 31.7 s, *p* < 0.05; 10 m/s 413.6 ± 66.7, *p* < 0.05; 11 m/s 357.7 ± 100.1 s, *p* < 0.05). Skull fractures and mortality were not observed at velocities below 11 m/s (Fig. 2B and C). Therefore, we selected 9 m/s as the test velocity for all our experiments. At 9 m/s, animals displayed a temporary discomfort as quantified by grimace scale analysis which was completely resolved 3 h after injury (Fig. 2D, one-way repeated measures ANOVA *F* = 21.379, *p* < 0.001 followed by Holm-Sidak vs. 30 min before injury).

**FIG. 3.**
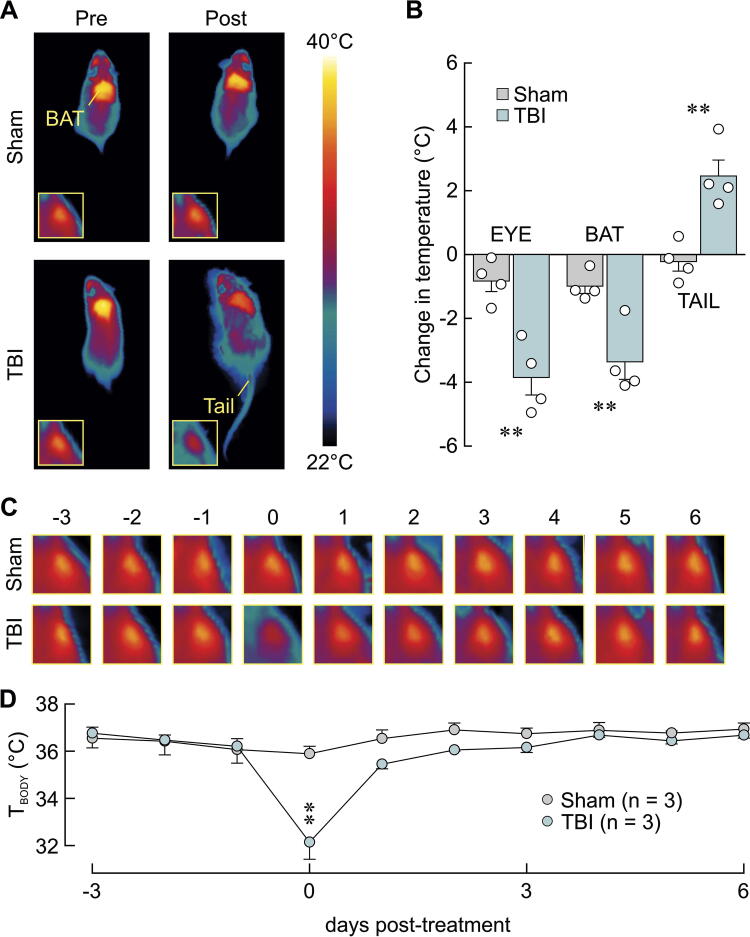
Lateral head impact induced temporary hypothermia. **(A)** Representative thermal images of a sham and a TBI mouse in the 45 min before (Pre) or after (Post) TBI/sham. Insets show eye of the same animal. **(B)** Lateral head impact decreased core body temperature as measured from eye temperature, brown adipose tissue temperature, and increased tail temperature. Change in temperature was calculated as maximal change relative to pre-TBI/sham values. Dots are individual values. **(C)** Representative daily eye thermal images of a sham and a TBI mouse. Numbers indicate days before or after TBI/sham. Temperature scale in **A** applies to **C**. **(D)** TBI-induced decrease in core body temperature (*T*_BODY_) resolved within 1 day after injury. **p < 0.01, *t*-test.

Hypothalamic injury is commonly associated with an acute defect in thermoregulation.^39^ We therefore examined the effect of TBI using infrared thermography (IT^40^). Indeed, compared with shams, TBI mice experienced a significantly greater decrease in core body temperature as assessed by IT of the eye^36^ (sham −0.8 ± 0.3°C, TBI −3.9 ± 0.5°C, *p* = 0.003, *t*-test) and a significantly greater decrease of BAT temperature (sham −1.0 ± 0.2°C, TBI −3.4 ± 0.5°C, *p* = 0.007, *t*-test; Fig. 3A, B). Moreover, mice subjected to TBI displayed a significantly greater increase in tail temperature (sham −0.2 ± 0.3°C, TBI 2.5 ± 0.5°C, *p* = 0.004, *t*-test; Fig. 3A, B), a feature indicative of vasodilation to dissipate body heat. Because recovery was not observed at this time scale, the experiment was repeated in another cohort of mice for which core body temperature was measured daily until 6 days post-TBI/sham. As illustrated in Figure 3C and D, significant TBI-induced hypothermia vs. sham was only present on the day of TBI/sham (sham 35.9 ± 0.3°C, TBI 32.1 ± 0.7°C, *p* = 0.009, *t*-test) and did not differ significantly from sham in the following days (*p* > 0.05, *t*-tests).

**FIG. 4.**
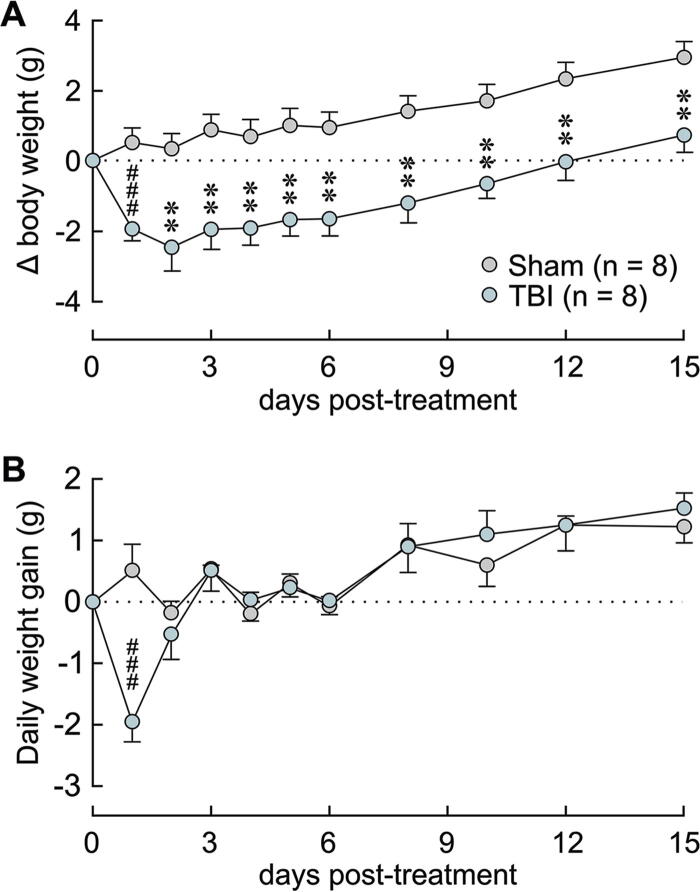
Lateral head impact induced temporary reduction in daily weight gain. **(A)** TBI caused an acute and sustained decrease in body weight. Change in body weight (Δ body weight) was calculated relative to body weight measured immediately before TBI/sham. **(B)** Daily weight gain, expressed as the change in weight relative to the previous day, was temporarily decreased by TBI. **p < 0.01, *t*-test. ^###^*p* < 0.001, Mann–Whitney.

Anorexia and weight loss represent another post-TBI symptom that may be associated with hypothalamic dysfunction. We therefore compared daily weight gain in sham and TBI-treated animals for 2 weeks. TBI caused an immediate and significant decrease in body weight compared with shams that was sustained throughout the 2-week period (Fig. 4A). Notably, this effect was associated with a significant decrease in daily weight gain on the first day following TBI compared with sham (sham 0.5 ± 0.4 g, TBI −2.0 ± 0.2 g, *p* ≤ 0.001, Mann–Whitney, Fig. 4B). However, daily weight gain was not different on subsequent days (*p* > 0.05; Fig. 4B).

**FIG. 5.**
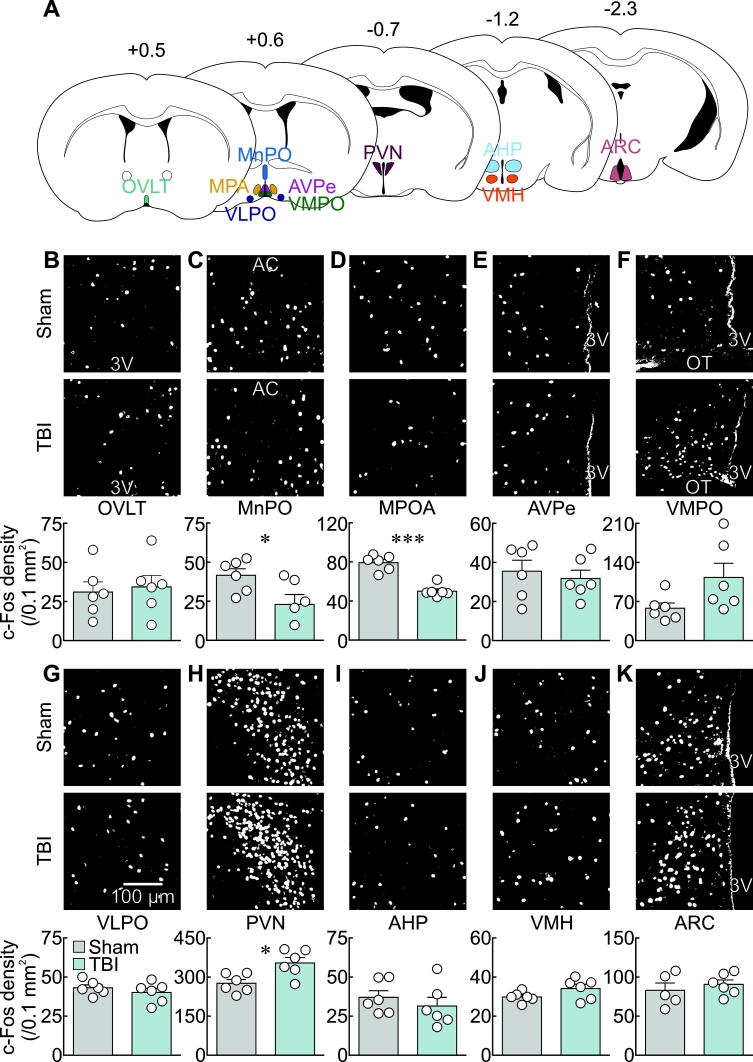
Lateral head impact activated the paraventricular nucleus and decreased c-Fos density in the median preoptic nucleus and medial preoptic area. **(A)** Schematic of regions of interest selected for c-Fos analysis. Numbers indicate anteroposterior position in mm relative to Bregma. (**B–K)** Representative c-Fos binary images of sham and TBI mice and c-Fos density quantification in the organum vasculosum lamina terminalis (OVLT, **B**), median preoptic nucleus (MnPO, **C**), medial preoptic area (MPOA, **D**), anteroventral periventricular area (AVPe, **E**), ventromedial preoptic area (VMPO, **F**), ventrolateral preoptic area (VLPO, **G**), paraventricular nucleus (PVN, **H**), anterior hypothalamic area (AHP, **I**), ventromedial hypothalamic area (VMH, **J**), and arcuate nucleus (ARC, **K**). Scale bar in **G** applies to all microscopy images. 3V, third ventricle; AC, anterior commissure; OT, optic tract. *p < 0.05, ***p < 0.001, *t*-test.

The findings above indicate that TBI delivered by lateral head impact at 9 m/s can cause acute yet reversible phenotypes commonly linked to hypothalamic function. The reversible nature of these features suggests that such phenotypes may result from alterations in neural activity rather than damage. We therefore compared expression of c-Fos in 10 different regions of the hypothalamus associated with regulation of body temperature and/or food intake 60 min after TBI or sham (Fig. 5A–K). We found significant TBI-induced decreases in c-Fos expression in the median preoptic nucleus (MnPO: sham 41.6 ± 4.2 nuclei/0.1 mm^2^, TBI 23.0 ± 6.3 nuclei/0.1 mm^2^, *p* = 0.03, *t*-test, Fig. 5C) and the medial preoptic area (MPOA: sham 79.3 ± 3.3 nuclei/0.1 mm^2^, TBI 50.1 ± 2.5 nuclei/0.1 mm^2^, *p* = 0.00003, *t*-test, Fig. 5D). Conversely, there was significant increase in c-Fos expression in the paraventricular nucleus (PVN: sham 276.1 ± 14.6 nuclei/0.1 mm^2^, TBI 354.2 ± 20.9 nuclei/0.1 mm^2^, *p* = 0.01, *t*-test, Fig. 5H).

## Discussion

Hypothalamic symptoms after TBI are commonly attributed to tissue damage within this structure.^23^ Indeed, persistent defects in thermoregulation, appetite control, and pituitary function are commonly linked to visible damage to hypothalamic tissue.^39,41–43^ A significant body of literature exists regarding the molecular mechanisms that underlie the secondary (inflammatory) expansion of tissue necrosis following TBI in many brain regions,^27^ including the hypothalamus.^23^ Such changes are consistent with the emergence of delayed and worsening symptoms in these patients.^13,44^ However, some phenotypes associated with TBI demonstrate rapid onset (minutes) and resolve within days or weeks^11,24^ and occur in the absence of observable tissue damage. Phenotypes such as these are presumably mediated by functional changes in neuronal activity that occur in the absence of cell death. How TBI can cause such changes is mostly unknown and defining their underlying mechanisms could improve our ability to alleviate these acute symptoms.

Two commonly used models of TBI involve controlled cortical impact (CCI) or fluid percussion injury (FPI).^45^ Although these techniques allow the production of highly reproducible tissue insults, they both require surgical exposure of the cerebrum and prolonged anesthesia which could indirectly affect hypothalamic functions.^46^ Moreover, these models only target small portions of the cortical surface and require head restraint, which precludes the occurrence of rotational acceleration. As such, CCI and FPI may not be ideally suited for the analysis of injury and dysfunction of deep structures such as the hypothalamus. Another popular TBI model, the weight drop method, also permits calibrated closed-head injuries. However, the impact required to produce desired phenotypes using this model is often accompanied by a high mortality rate.^45^ Moreover, because the animal’s head movement is typically restricted by a cushioned surface, TBI delivered by weight drop may underemphasize the head acceleration typically associated with injuries in humans. Indeed, modelling studies show that mechanical perturbation of the hypothalamus during TBI may be exacerbated by rotational acceleration.^47^ In order to circumvent these limitations, our study adapted a lateral head impact model that was specifically designed to approximate the biomechanics of human TBI.^34^ In our application, a single impact was delivered and velocity was adjusted to prevent skull fractures, overt tissue damage and chronic pain. We therefore believe this model reflects a mild form of TBI.

A hallmark of hypothalamic dysfunction is perturbed thermoregulation.^48^ TBI-induced hyperthermia is commonly attributed to the production of a fever resulting from neuroimmune inflammatory processes,^49^ whereas hypothermia is attributed to hypothalamic damage.^50^ In our experiments, TBI caused a profound acute drop in body temperature that recovered within 48 h, which was presumably caused by a reduction in heat generation by BAT coupled to an increase in heat loss owing to tail vasodilation. These observations suggest the involvement of a temporary and reversible inhibition of hypothalamic body heating mechanisms. Indeed, c-Fos analysis revealed an acute suppression of activity in the MnPO and MPOA. Although these nuclei are well known to contribute to thermoregulation,^51^ additional studies are required to establish the network and functional basis of hypothermia following lateral head impact. Interestingly, a previous study in rats has shown that TBI can induce a sequence of hypothermia and hyperthermia followed by recovery.^52^ Our results suggest that acute hypothermia may reflect an immediate mechanically induced disruption of hypothalamic function, whereas the hyperthermic phase may reflect a delayed inflammatory response associated with a secondary effect of the injury. A previous study has shown that repetitive mild TBI delivered to rats reduced the amplitude of circadian rhythms in body temperature and locomotor activity.^53^ Notably, repeated mild TBI was found to affect the suprachiasmatic nucleus, a structure which serves as the body’s master circadian clock, and the authors concluded that circadian phenotypes reflected hypothalamic damage. Future experiments should determine if circadian rhythms can be reversibly affected by mechanical activation of this part of the hypothalamus.

A disruption of food intake is commonly associated with TBI^18,54^ and case reports have indicated eating disorders may persist for years following TBI,^19,55^ consistent with irreversible damage to the hypothalamus.^56^ Although some patients can display increased or decreased weight months following TBI, others recover their normal weight suggesting that some TBI-induced eating disorders are reversible.^18^ Indeed, our results show that lateral head impact causes an acute loss of daily weight gain, which resolves within 24 h. This phenotype presumably reflects the absence of food intake during the day following TBI which is also consistent with a temporary dysfunction of the hypothalamus. Indeed, our c-Fos analysis shows activation of the PVN, and previous studies have shown that excitation of PVN oxytocin neurons can reduce food intake.^57^ In contrast, no changes in c-Fos expression were observed within the arcuate nucleus, another structure involved in the control of food intake. Additional studies are required to define the basis for reversible anorexia following TBI.

The PVN is a hypothalamic hub involved in the control of many other homeostatic functions including stress, reproduction, growth, sympathetic outflow, and osmoregulation.^58^ Therefore, many additional phenotypes may also be induced by our model of TBI and should be investigated in future studies. In particular, examining c-Fos expression within chemically defined subsets of neurons may yield significant differences that may have been missed by our global analysis. For example, the arcuate nucleus is known to contain neurons that either promote or suppress food intake. Therefore, possible changes in c-Fos expression in these subtypes should be analyzed separately. Moreover, our c-Fos analysis indicated trends toward increased c-Fos expression in the VMPO, a structure known to contribute to the regulation of body temperature and sleep wake rhythms, among other functions.^59^ A more exhaustive analysis of c-Fos expression in this and other hypothalamic nuclei is therefore warranted together with an in-depth study of functionally related phenotypes. It will also be of interest to explore changes in hypothalamic phenotypes induced by TBI as a function of age. The present experiments did not include aged mice; they were performed on animals ranging from 2 to 4 months old. Nonetheless, this is an age range which features substantial brain development and approximates the transition between adolescence and young adulthood in humans.^60^ Although the average time-to-right was not different between mice aged 2–2.5 months old (250 ± 30 s; *n* = 15) and mice aged 3.5–4 months old (237 ± 47 s; *n* = 12; *p* = 0.806, *t*-test), it will be important to examine if other phenotypes differ at these time points in future experiments.

A motivating factor for this study was the hypothesis that a mechanical activation or inactivation of neurons within the hypothalamus could result from mild TBI and thus produce acute, reversible phenotypes in contrast to those associated with irreversible tissue damage. Our findings provide strong support for this hypothesis and suggest additional work should be performed to identify the mechanosensitive elements (ion channels and specific neurons) that may be involved in producing acute and reversible dysfunctions following TBI.

## Conclusions

Our study shows that a single lateral head impact calibrated to produce loss of consciousness in the absence of overt tissue damage, skull fractures, or chronic pain can produce acute, reversible phenotypes typically associated with dysfunction of the hypothalamus in mice. c-Fos analysis revealed possible functional changes in the activity of various groups of neurons and suggests that mild hypothalamic phenotypes can be caused by temporary, mechanically induced changes in neural function without irreversible damage.

## Abbreviations Used

3V: third ventricle
AC: anterior commissure
AHP: anterior hypothalamic area
ARC: arcuate nucleus
AVPe: anteroventral periventricular area
BAT: brown adipose tissue
CCI: controlled cortical impact
FPI: fluid percussion injury
IT: infrared thermography
MnPO: median preoptic nucleus
MPOA: medial preoptic area
OT: optic tract
OVLT: organum vasculosum lamina terminalis
PBS: phosphate-buffered saline
PVN: paraventricular nucleus
TBI: traumatic brain injury
VLPO: ventrolateral preoptic area
VMH: ventromedial hypothalamic area
VMPO: ventromedial preoptic area
ZT: Zeitgeber time
